# The Effect of Higher Education on Probationers' Salaries

**Published:** 1919-02-01

**Authors:** 


### THE MATRONS' AND SISTERS' DEPARTMENT.

### THE EFFECT OF HIGHER EDUCATION ON PROBATIONERS' SALARIES.

The old plan of using probationers as a form of cheap labour and letting them learn what they could in the process of performing mechanical tasks has gone, never to return. Every movement in the direction of a higher curriculum tends to release them more surely from the status of drudges and ratify their status as pupils. Now the difference between a drudge and a pupil lies in this that the pupil recognises an obligation to pay while the drudge insists upon her claim to be paid. The pupil may quite reasonably be allowed to pay for the advantages received in services by surrendering part of her time to active labours. Such service rendered in lieu of fees, was a recognised feature of university education in former days and since none can profitably devote more than a few hours a day to study, it answered very well. In the case of nurse pupils this system is eminently fair because practice in the manual and partly mechanical duties they are called on to perform early in their training is essential to the mastery of their profession. Like engineers, they are no good at all if their training is purely theoretical. They must go through the workshops and master every detail of work by doing it till they do it as well as it can be done.

We must be on our guard in this matter. The training-schools want a choice of the best material which is available, and therefore they must never resort to the old-fashioned plan of asking a premium from their probationers and thus limiting the supply to women with private means. But they also offer every day more and more privileges to their pupils, and hence they need not compete with mechanical occupations by offering substantial salaries to unskilled workers. Training-schools whose certificates do not open up a good career may still be obliged to tempt young women to enter their gates. It seems reasonable that such hospitals should pay more for their pupils, since by beginning in a very small or special hospital a woman prolongs her pupil stage and postpones that in which her earning capacity is at its zenith. But the well-equipped training-schools already give so substantial a return for the simple labours which the probationer has to perform, that it is straining their resources too far to expect them to add what may be called a living wage in addition.

Has any one noticed that the authorities of the Leeds General Infirmary estimate the annual cost per head in addition to salary of the forty new nurses whom they are adding to their staff at £80? This is, of course, at war prices, but even when the cost of food is considerably reduced the figure will still be something like 30s. a week. We could wish that the monetary value of the emoluments offered to probationers were carefully worked out in all institutions and fully stated in the forms of application which probationers are required to fill in.

The present scarcity of good candidates due to the overwhelming demand for unskilled workers already shows symptoms of lessening. Within a couple of years it may be safely predicted that the competition to enter good nurse-training schools will be keener than ever. The far-sighted policy of the College of Nursing will then meet with its reward. Higher grade teachers will attract higher grade women. Nursing sisters, no longer cramped by rigid conformity to a semi-conventual rule, but with faculties shaped to national ends, will be available to carry the good methods into smaller institutions.

Thus, in proportion as higher education tends to improve the class of candidate and draw women into the nursing profession who aim at developing their faculties for the public good, it will make probationership a privilege to be sued for and bring about once more a rich choice for the selecting matron. We deem it very doubtful whether the present rate of pay extended to utterly untrained women will continue. There can hardly be a doubt it has reached its maximum. If the present costly style of establishment required for the training-school is to be maintained, and few will contend against this, beginners ought to be content with finding their every want anticipated during their early training days and receiving a pocket-money allowance in lieu of salary.

